# Millimetre-wave comb generated by an optical microcomb

**DOI:** 10.1038/s41467-026-76747-2

**Published:** 2026-08-20

**Authors:** L. Peters, A. Cutrona, A. R. Cooper, L. Olivieri, F. Getman, V. Cecconi, N. Paul, D. Das, M. Rowley, S. T. Chu, B. E. Little, R. Morandotti, D. J. Moss, J. S. Totero Gongora, A. Pasquazi, M. Peccianti

**Affiliations:** 1 Emergent Photonics Research Centre (EPicX), Dept. of Physics, Loughborough University, Loughborough, UK; 2 Emergent Photonics Lab (EPic), Department of Physics and Astronomy, University of Sussex, Brighton, UK; 3 Department of Physics, City University of Hong Kong, Hong Kong, SAR China; 4 QXP Technologies, Inc., Xi’an, China; 5 INRS-EMT, Varennes, Québec Canada; 6 Optical Sciences Centre, Swinburne University of Technology and ARC-COMBS, Hawthorn, VIC Australia

**Keywords:** Terahertz optics, Solitons

## Abstract

Millimetre-wave baseband combs are relevant to wireless links, sensing, and device characterisation. Direct broadband generation from an optical microcomb has remained elusive because of challenges in photoconductive conversion. Here, we demonstrate a 50 GHz repetition-rate millimetre-wave baseband comb covering the sub-THz region, generated by photoconductive conversion of a laser-cavity-soliton microcomb. The microresonator-filtered source combines low free-running phase noise and high spectral efficiency, enabling coherent conversion without additional optical amplification to single-cycle, carrier-envelope-offset-free waveforms with multiple harmonics directly observed in the converted output. The source supports free-running time-domain spectroscopy over optical path delays above 8 m, and its lines are driven by the optical repetition-rate harmonics with traceable improvement of synchronisation performance upon external locking. In addition, multisoliton operation reshapes the harmonic envelope while preserving the common line spacing, providing a simple route to source-level spectral control in the sub-THz region.

## Abstract (web summary)

This study demonstrates a millimetre-wave baseband comb generated directly from an optical microcomb, enabling coherent sub-THz waveforms and source-level spectral control.

## Introduction

The development of high-stability millimetre-wave and terahertz (THz) baseband sources is important for applications in communication^1–5^, sensing^3,6–11^, and timing and positioning^12–19^. The next generation of millimetre-wave (mm-wave) frequency standards in the sub-THz range will form the foundation of future 6G communication systems and positioning devices^4,5,13^. It will require precision time-traceability^20,21^ and low phase noise^9^. Over the past two decades, mm-wave sources have become progressively linked to photonics and, in particular, to optical frequency combs^22–24^, which presently enable the most advanced optical metrological systems, both in the spectral and time domains^22–24^. This includes optical frequency combs that can generate ultralow-noise single frequencies in the mm-wave baseband regime. Prominent related examples are quantum cascade lasers^25–30^ and electronic oscillators^31–33^, which dramatically improve spectral purity and frequency accuracy when stabilised using optical frequency combs. These hybrid systems are particularly important for spectroscopy applications, including the interrogation of quantum states^8,34^, time-domain THz spectroscopy^35^, and dual-comb schemes^36–38^. More broadly, optical frequency combs that can be utilised to generate baseband mm-wave signals are expected to be a critical building block of next-generation wireless communications and positioning systems, both as communication sources, e.g., as multichannel solutions for parallel frequency-division multiplexing and multi-user links or as calibration and metrological devices^32,37,39^. Compatibility with coherent optical links^13,20^, especially for clock-stabilised frequency distribution, is a key feature. In this scenario, microcomb-based mm-wave sources^14,16,17^ have demonstrated compactness and unprecedented spectral purity, especially when the microresonator combs^14,16,17,40–50^ are operated in the so-called soliton regime^51–53^. Because microcombs are based on monolithic, integrated resonators, they have intrinsically superior phase noise performance, particularly in their repetition rate, which naturally falls in the sub-THz range. This specific combination of features makes microcomb-driven mm-wave generation highly desirable compared to solutions based on traditional mode-locked pulsed lasers^54–57^. Recent demonstrations^58,59^ achieved ultra-low phase noise in the microcomb repetition rate that was efficiently transferred to the mm-wave domains, enabling the creation of ultra-high-purity carriers. Here, the excellent phase noise achieved in the optical regime was moved to lower frequencies via optical division^15–19^, producing state-of-the-art high-purity, single-frequency microwave sources^21,58,60–64^.

Beyond single-frequency generation, the next major frontier is the direct generation of a broadband coherent frequency comb in the sub-THz regime, important for channel-multiplexed communications, broadband spectroscopy, and the precision evaluation of emerging 6G devices^1–5^.

**Fig. 1:**
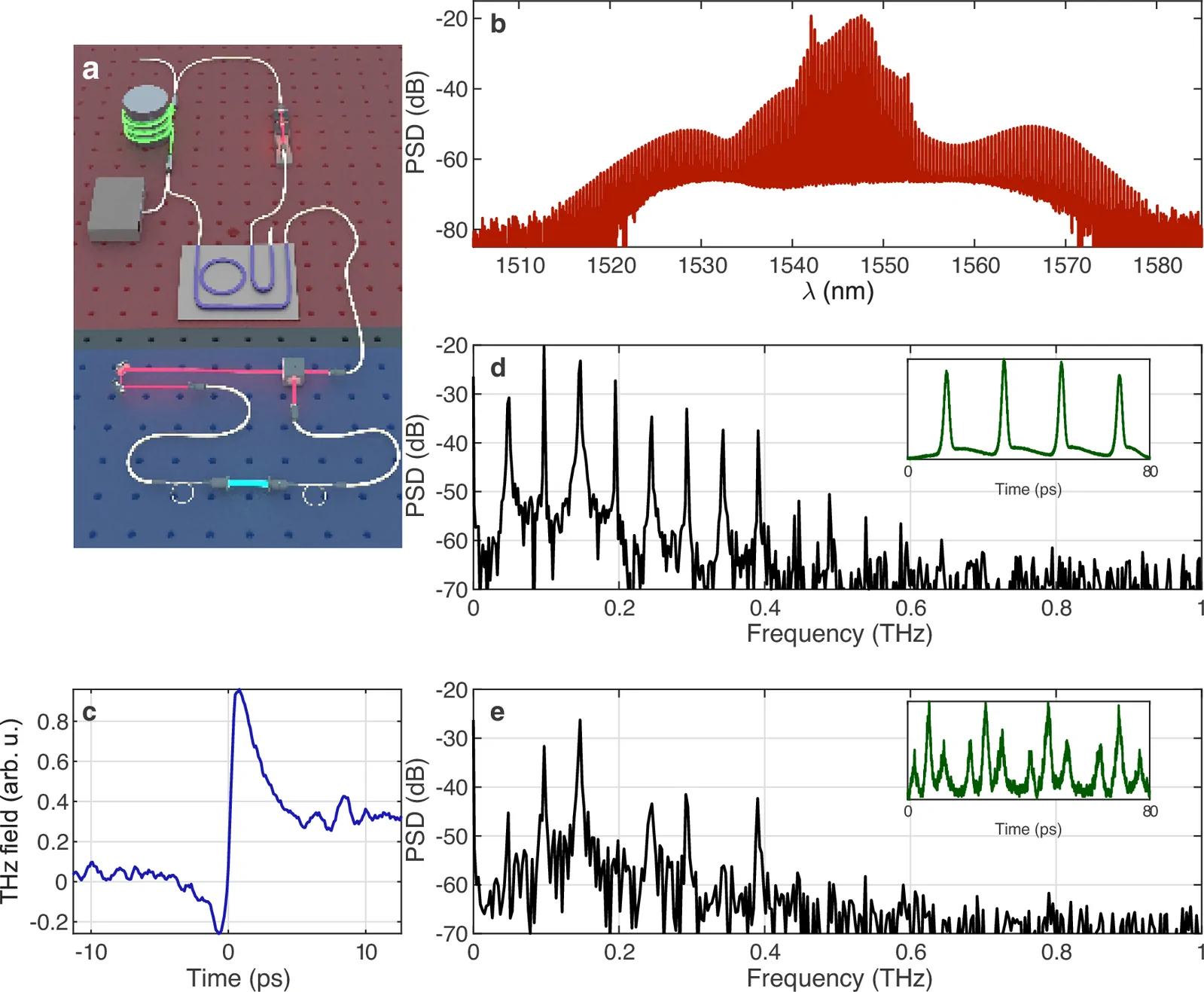
Generation of a millimetre-wave comb via photoconductive conversion of a laser cavity-soliton microcomb. **A** microcomb laser, consisting of a nonlinear microresonator (FSR 48.9 GHz) filtering a fibre laser cavity loop (FSR ~ 95 MHz) (red region), is coupled to a time-domain spectroscopy (TDS) system (blue region) via a beam splitter feeding both transmitter and receiver photoconductive-switch antennas. A mechanical stage controls the relative pulse delay between the pulse replicas. **b** A typical optical spectrum of a single-soliton microcomb state. **c** TDS trace of a single-cycle millimetre-wave pulse obtained by feeding the TDS setup with a single-soliton microcomb (roughly 5 mW average power), without further processing or amplification. The slight pedestal variation in the time-domain trace is attributed to system drift during mechanical scanning. **d** Normalised Fourier amplitude spectrum in dB (arbitrary units) of the millimetre-wave trace from c, highlighting the comb structure; the inset shows the autocorrelation. **e** Same as (**d**), but for a state composed of two non-equidistant solitons, showing preservation of the common comb spacing together with a redistributed harmonic envelope.

Here, we demonstrate direct generation of a broadband millimetre-wave baseband comb in the sub-THz regime from an optical microcomb. In contrast to previous microcomb-based mm-wave approaches, which have primarily targeted single-frequency carriers through optical division, photodiode-based generation, or selected-frequency photomixing^14,16,17,37,55,58,60,65^, the present work accesses a directly observed broadband baseband comb with multiple mutually coherent harmonics in the converted output. Photoconductive time-domain generation and sampling are instrumental in this context because they provide direct access to single-cycle, carrier-envelope-offset-free waveforms in a TDS-style configuration, rather than only to a narrowband carrier. A key enabling aspect of the present implementation is the use of a microresonator-filtered fibre laser operating in the laser-cavity-soliton regime (Fig. 1a, b), whose high spectral efficiency, i.e., the distinctive lack of a strong non-pulsed background for the specific soliton state, makes direct coupling to photoconductive emitters practical, including operation without additional optical amplification (Fig. 1c). We further show that multisoliton states provide a simple source-level mechanism for reshaping the harmonic envelope of the generated mm-wave comb while preserving the common line spacing (Fig. 1d, e).

The capabilities demonstrated here motivate several realistic application directions. Direct baseband comb generation with multiple harmonics is relevant to broadband calibration and multi-tone device characterisation; long-delay coherence is relevant to relaxed-synchronisation TDS architectures; and source-level spectral redistribution through multisoliton states is relevant to multi-channel spectroscopy and parallel mm-wave links. In this sense, the present work establishes a microcomb-compatible route to broadband photoconductive mm-wave comb generation.

## Results and discussion

A schematic of the experimental setup is presented in Fig. 1a. The microcomb laser^66,67^ is based on a nonlinear microresonator closed in an erbium-doped fibre-amplifier (EDFA) cavity. A typical laser-cavity-soliton microcomb produced by the system is shown in Fig. 1b, with a 48.91 GHz repetition rate. The millimetre-wave comb source/detection system is based on commercially available THz-TDS components. It is fed directly by the laser-cavity-soliton pulses produced at the resonator output (see Methods for details). The TDS setup comprises two commercial photoconductive antennas fed by two output replicas delayed by a mechanical optical delay line. Specifically, a photoconductive antenna generates a millimetre-wave waveform *E*_*THz*_(*t*) that follows the derivative of the optical intensity profile *I*(*t*), feeding it^68^:

1 $${E}_{THz}(t)\propto \frac{\delta I(t)}{\delta t}.$$

The optical pulse envelope also serves as the sampling function in the TDS receiver, and the detected field is the convolution of the emitted THz transient with the sampling-pulse envelope, as written below (further details in the “Methods” section):

2 $${E}_{THz}^{det}(t)={E}_{THz}(t)*I(t).$$

**Fig. 2:**
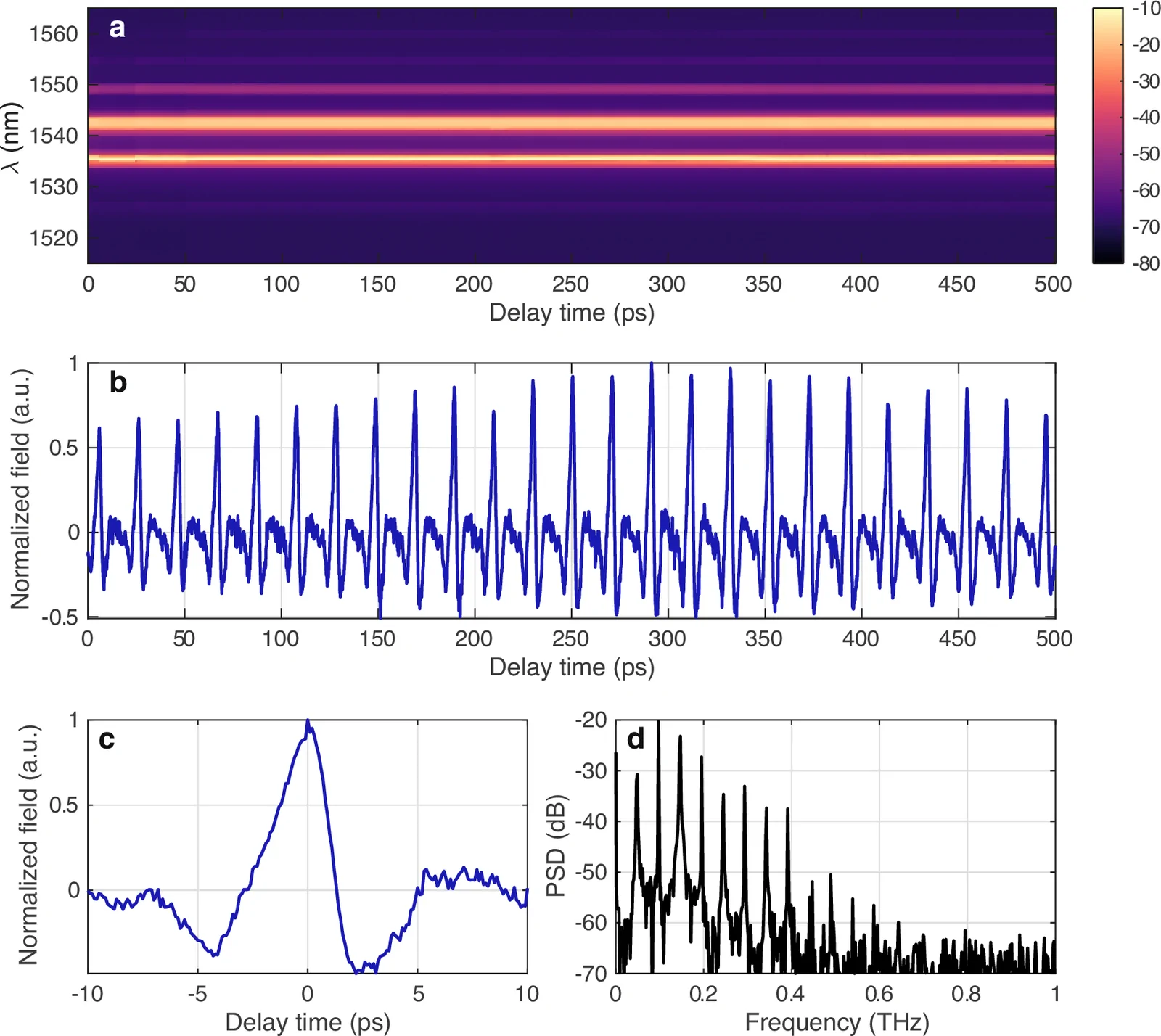
Long-term stability of millimetre-wave generation for single-soliton states. **a** False-colour map, with colour scale in dB, of the optical-spectrum-analyser traces acquired during the TDS scan, showing wavelength versus time-delay step. **b** TDS trace obtained by converting the microcomb pulse extracted at approximately a time delay 290 ps. **c** Single-cycle trace extracted from (**b**). **d** Normalised Fourier amplitude spectrum in dB (arbitrary units) of the waveform in (**b**).

**Fig. 3:**
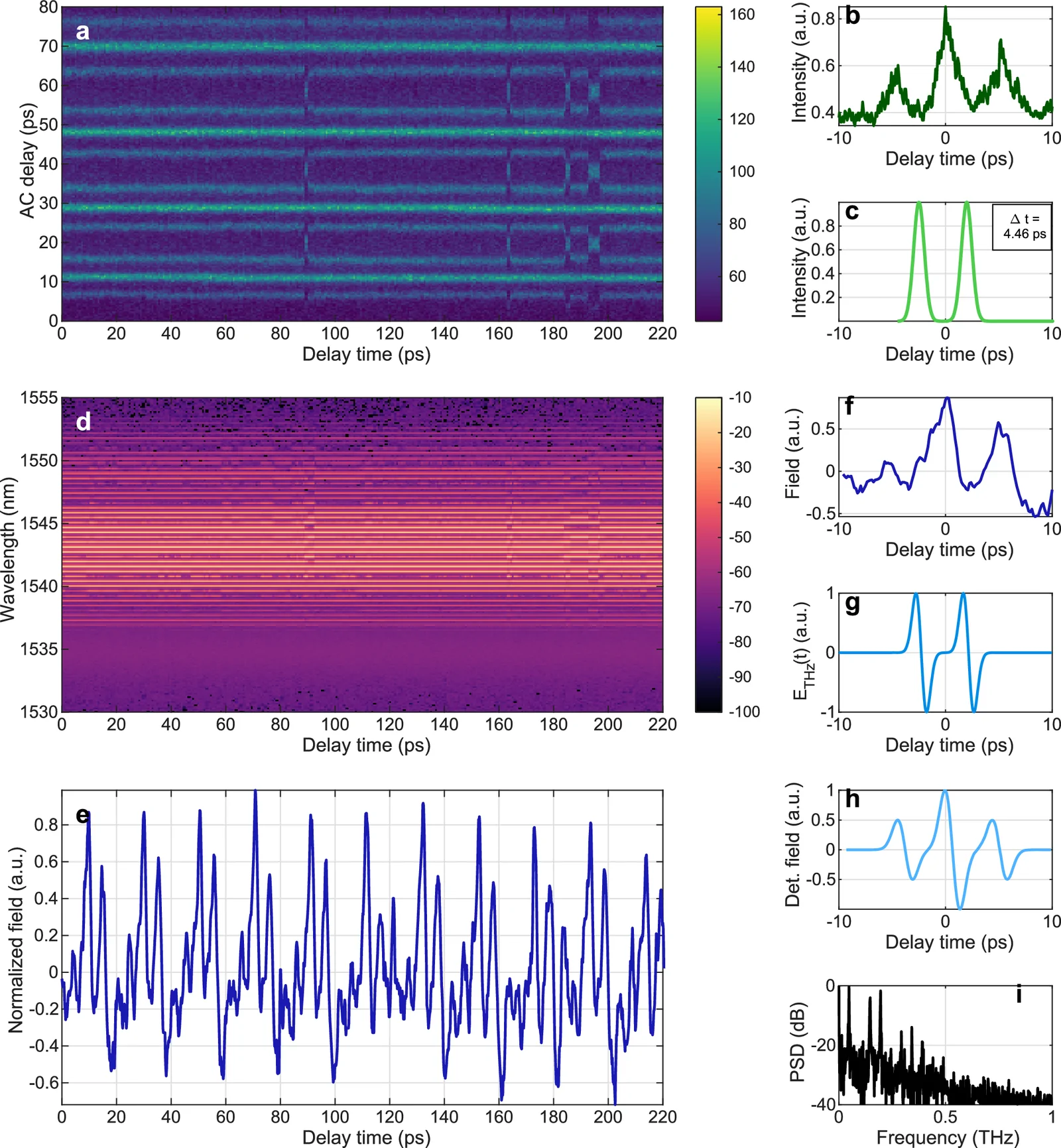
Long-term robustness and high-signal-to-noise ratio time-domain spectroscopy for a state with two non-equidistant solitons. **a** False-colour map in dB on the colour axis of the autocorrelation traces in time. **b** A typical autocorrelation trace at *t*  = 0. **c** Reconstructed double soliton state with periodicity 20 ps and soliton spacing 4.46 ps extracted from the autocorrelation trace. **d** False-colour map of the optical spectrum analyser traces in time. **e** Experimental terahertz time-domain spectroscopy trace. **f** Experimental TDS trace for a single period. **g** Expected generated waveform converting with Eq. (1) the optical pulses in (**c**). **h** Reconstructed TDS trace using Eq. (2) with the generated waveform in g and the optical pulses in (**c**). **i** Experimental THz spectrum.

Figure 1c shows the conversion of a free-running, single-soliton case, with about 5 mW output power, used to feed the two photoconductive switches without any optical post-processing. The free-running microcomb laser consumes only the electrical power necessary to run the 980 nm EDFA pump, which is below 1.5 W in this configuration. Although the measurement required several seconds of averaging per data point (see “Methods” for full details), this demonstrates the main compatibility feature of our platform with commercial photoconductive THz systems. The pulse profile is remarkably single-cycle, well reproducing a differential pulse as expected by Eq. (1), with a peak-to-peak duration (half-cycle) on the order of 1.5-2 ps. Such a duration is consistent with the general output pulse width of the comb (roughly 500 fs). It is important to stress how, in our realisation, the limits imposed by off-the-shelf solutions play out. Indeed, commercial THz photoconductive switches (as the ones we utilised) are designed as resonant antennas for standard pulsed lasers with repetition rates (~ 100 MHz) much lower than the ~ 50 GHz of our microcomb source and exhibit reduced conversion efficiencies outside the sub-THz region, highlighting a tremendous potential for improvement in efficiency. To provide comprehensive diagnostics and overcome such limitations, we filtered and amplified the comb to increase the signal-to-noise ratio (SNR); see Methods for details. Examples of normalised Fourier amplitude spectra extracted for the single-soliton (Fig. 1d) and two-soliton (Fig. 1e) cases show the comb structure of the generated mm-wave signal. In the single-soliton case, Fig. 1d directly reveals a set of harmonics spaced by the microcomb repetition rate. In the two-soliton case, Fig. 1e shows that the common spacing is preserved, while the harmonic envelope is redistributed by interference between the delayed soliton pulses. This source-level spectral reshaping is analysed in greater detail in Figs. 2, 3.

Figure 2 provides an overview of the system performance in the single-soliton regime. Figure 2a shows the long-term behaviour of the microcomb, displaying the optical spectrum recorded over roughly one hour. The TDS field trace was reconstructed over a temporal window exceeding 500 ps, covering approximately 25 pulses at the microcomb repetition rate of ~ 50 GHz. Notably, although TDS scanning is a typically slow process, the LCS microcomb demonstrated high stability throughout the scan (around 6000 samples with a second-scale lock-in amplifier integration time), reflecting its inherent self-recovery nature^66,67^. Figures 2a, b both illustrate the consistent spectral properties tracked during the scan, with the TDS trace (Fig. 2b) exhibiting only long-term DC drifts. The pulse field profile, zoomed in Fig. 2c, is typical for photoconductive-based TDS^68^ and resembles a pulse derivative (Ricker wavelet). The corresponding normalised Fourier amplitude spectrum reveals a measurable bandwidth exceeding 600 GHz within our detection dynamics, with ten resolvable harmonics of the ~ 50 GHz repetition rate and good coverage of the sub-THz region.

We then explored the capability of the system to control and tune the millimetre-wave comb emission. Multisoliton states generally manifest as non-equidistant series of pulses, and spectral shaping and control based on soliton crystals is well-studied in the microcomb literature^69,70^. The simplest scenario is that of a two-soliton state, where the spectrum depends directly on the relative delay between the two soliton pulses. The results shown in Fig. 1e demonstrate a markedly different spectral distribution compared to the single-soliton state. Figure 3 offers a more detailed analysis of a two-soliton state, visible in the autocorrelation traces (Fig. 3a for the temporal evolution, Fig. 3b for a single trace, and Fig. 3c for the reconstructed intensity profile), with a relative delay of 4.46 ps-approximately 22.3 % of the period. Multisoliton states tend to pin to defects, preserving the configuration if not strongly perturbed. The state remained stable throughout the 30 min scan (as seen in both the autocorrelation evolution, Fig. 3a, and the OSA traces, Fig. 3d, producing a uniform millimetre-wave waveform in Fig. 3e. Examining a single period in Fig. 3f, we observe three pulses, with a central dominant peak. The numerical waveform in Fig. 3g, obtained by differentiating the pulse train in Fig. 3c, represents the effective generating field created by the double soliton state. The TDS system, however, retrieves its convolution with the optical pulse train in Fig. 3c, which is shown in Fig. 3h, following Eq. (2), and aligns well with the experimental TDS trace. The normalised Fourier amplitude spectrum in Fig. 3i, obtained from the detected TDS data, shows the characteristic signature of interference. The reduced visibility of some harmonics in this detected spectrum is not suppression at the source. Rather, it arises from the temporal convolution intrinsic to the TDS detection process: the emitted field is sampled by the optical pulse train, so the detected spectrum is filtered by the finite temporal structure of the sampling pulses, as illustrated by the model in Fig. 3h and Supplementary Fig. 2. Supplementary Fig. 1 shows a similar case for a different pulse delay, further demonstrating that simple control of the inter-soliton delay enables reconfiguration of the harmonic envelope while preserving the common comb spacing.

**Fig. 4:**
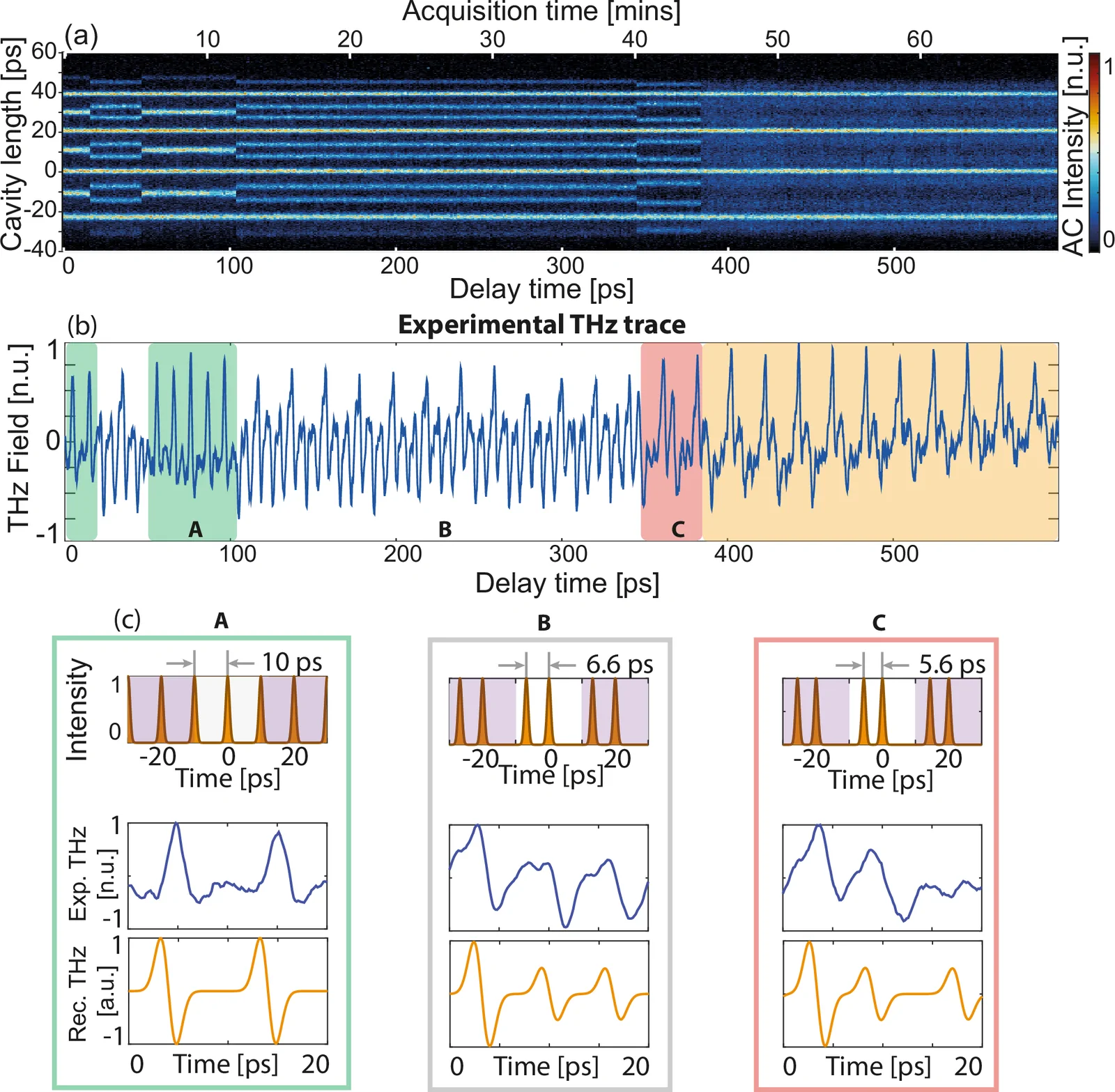
Terahertz generation from evolving soliton states. **a** Experimental autocorrelator evolution map showing transitions between soliton states over the acquisition time. **b** Experimental terahertz time-domain spectroscopy (TDS) trace, highlighting the distinct THz responses corresponding to different soliton configurations: A - double soliton with equal spacing (*τ *= 10 ps, green), B - double soliton with *τ *= 6.6 ps (grey), C - double soliton with *τ *= 5.6 ps (red), and - single soliton (orange). **c** Insets A–C : For each configuration, the reconstructed optical intensity profile (top), experimental THz trace (middle), and simulated THz trace (bottom) are shown.

In Fig. 4, we illustrate different soliton dynamics of the LCS during a very long TDS scan, where the laser is left under environmental perturbations and independently transitions between states. Interestingly, by positioning at the boundary between a single-soliton region and a multisoliton state (typically linked with high gain and main cavity detuning) in the parameter landscape, we can simply let the system drift with relatively loose temperature stabilisation to observe these transitions. Even under these conditions, the system shows remarkable stability over several tens of minutes, and the transitions are quite distinct and are triggered by substantial environmental fluctuations. We tracked the autocorrelation evolution during the scan (Fig. 4a), which reveals transitions from fundamental solitons to dual-soliton states and soliton molecules. Figure 4b shows the reconstructed mm-wave field. Three distinct two-soliton states are identified: Region I is an equally spaced two-soliton state (with a relative delay of 10 ps between pulses), Region II is unequally spaced (with a relative delay of 6.6 ps), and Region III is also unequal (with a relative delay of 5.6 ps). In Region IV, the system transitions to a single-soliton state. The insets in Fig. 4c compare the measured pulse field dynamics (blue plot) with the one estimated from the autocorrelation and a simple bipolar impulse response of the antenna (yellow plot), confirming the interpretation of the field dynamics as the signature of each state. It is evident that the THz emission is affected by the soliton dynamics, with noticeable changes in both phase and spectral content corresponding to each transition.

**Fig. 5:**
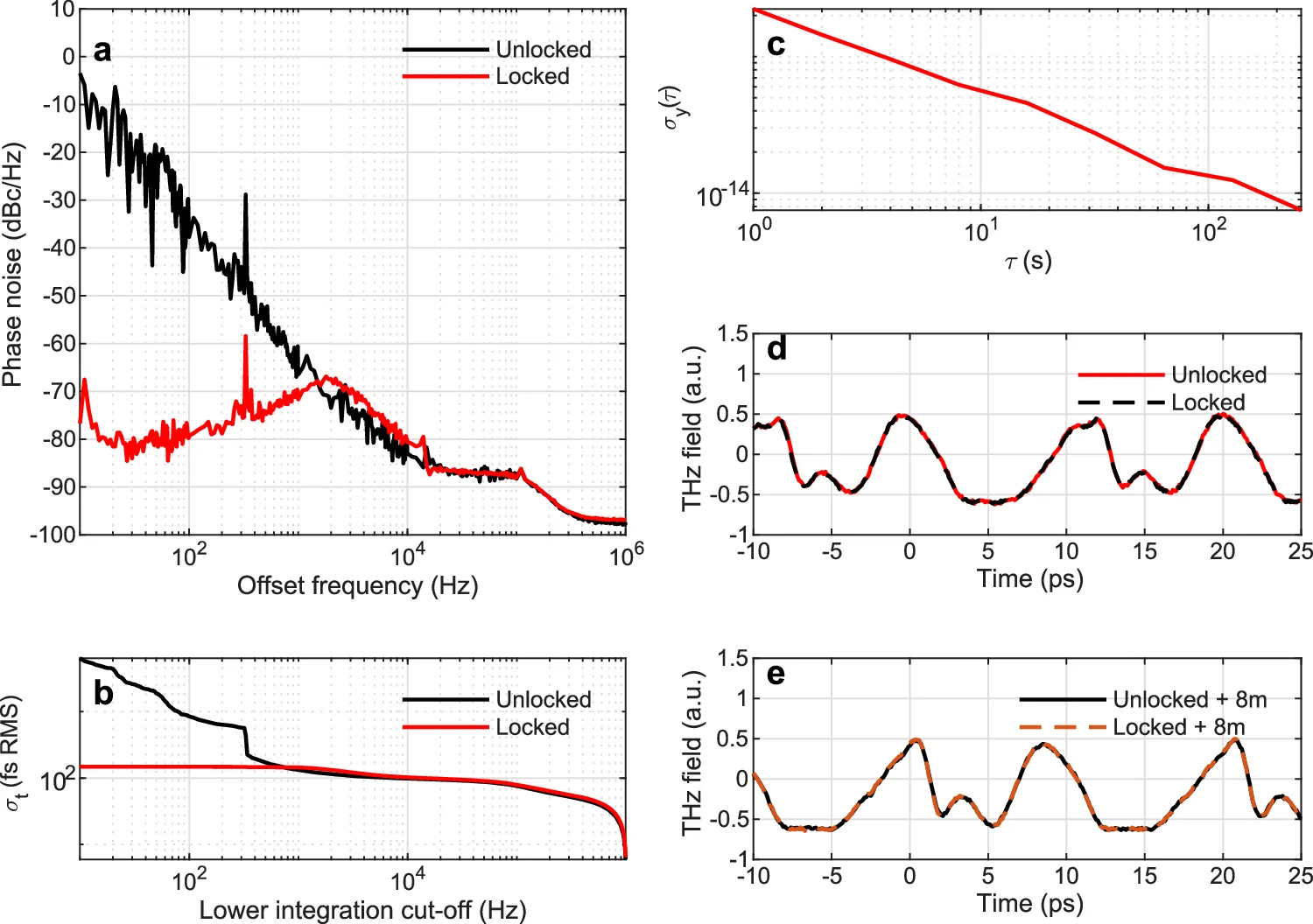
Referenced repetition-rate stabilisation and long-delay coherence. **a** Phase noise of the unlocked (black) and repetition-rate-locked (red) laser-cavity-soliton system. **b** Integrated timing jitter for the locked (red) and unlocked (black) cases. **c** Allan deviation of the referenced repetition-rate readout for the locked system. **d** Time-domain millimetre-wave field comparison of the unlocked (black) and locked (red) cases. **e** Same as (**d**), but for a TDS measurement where the detection antenna is fed with a pulse replica delayed by 8 m relative to the generating antenna.

Figure 5 explores how stabilising the GHz repetition rate of our laser-cavity-soliton microcomb influences the optical source and the reconstructed sub-THz TDS measurements. The phase noise of the free-running ~ 50 GHz repetition rate is depicted in black in Fig. 5a. In this case, the integrated timing jitter (Fig. 5b), evaluated from 1 MHz down to 10 Hz, is ~ 6.3 ps, but it is strongly affected by a specific low-frequency oscillation at ~ 400 Hz; in the range 600 Hz–1 MHz, the jitter remains well below ~ 150 fs (Fig. 5b). This specific noise contribution can be easily stabilised with mechanical methods, which we applied in our system using a piezo-stretcher on the fibre cavity (see “Methods”), locking the repetition rate to a microwave GPS-disciplined reference via electro-optic downconversion.

After disciplining the source (phase noise in Fig. 5a, red curve), it strongly improves long-term stability. The high-frequency contribution between 100 kHz and 1 MHz changes only from ~ 79 fs to ~ 75 fs. The lock, therefore, does not alter the short-term performance. The integrated jitter falls below ~ 150 fs in the full range, showing that the low-frequency portion of the noise is almost completely removed; this is even more visible in the Allan deviation shown in Fig. 5c. It is important to note that, in our measurement, both the repetition-rate downconversion scheme and the locking electronics are referenced to the same GPS-disciplined source (see “Methods”). Accordingly, the Allan deviation in Fig. 5c is interpreted as the referenced stability of the repetition-rate readout down to the known stability of our GPS-disciplined reference, at the level of a few parts in 10^−12^ over averaging times from 1 to 100 s. Below that level, it should be interpreted only as the transfer coherence of the locked comb/readout chain, and hence as a measure of locking quality rather than an independent absolute stability measurement.

The stabilisation has no observable effect on the millimetre-wave comb TDS trace, including output power, spectral shape, or conversion efficiency, as shown in the example in Fig. 5d. Pulse replicas remain mutually coherent over the approximately 500 ps TDS delay scan window in the free-running case. In other words, the TDS reconstruction does not require the generation and detection paths to be aligned to a unique zero-delay condition. In our system, we verified accurate THz waveform reconstruction for optical path differences as large as 8 m between the pulse replicas feeding the generating and detecting antennas, corresponding to about 27 ns and over 1000 pulse periods. Over such delays, we detected no statistically meaningful deviation in the mm-wave spectrum; any residual amplitude changes are attributed to power drift (Fig. 5e and Supplementary Fig. 3), where Supplementary Fig. 3 shows the corresponding amplitude spectra for Fig. 5d, e. The free-running comb, therefore, remains sufficiently stable for practical spectroscopy over multi-metre delays, while repetition-rate locking-common in optical-frequency-comb metrology, can be applied to this mm-wave platform. In this sense, the results show compatibility with precision frequency control.

**Fig. 6:**
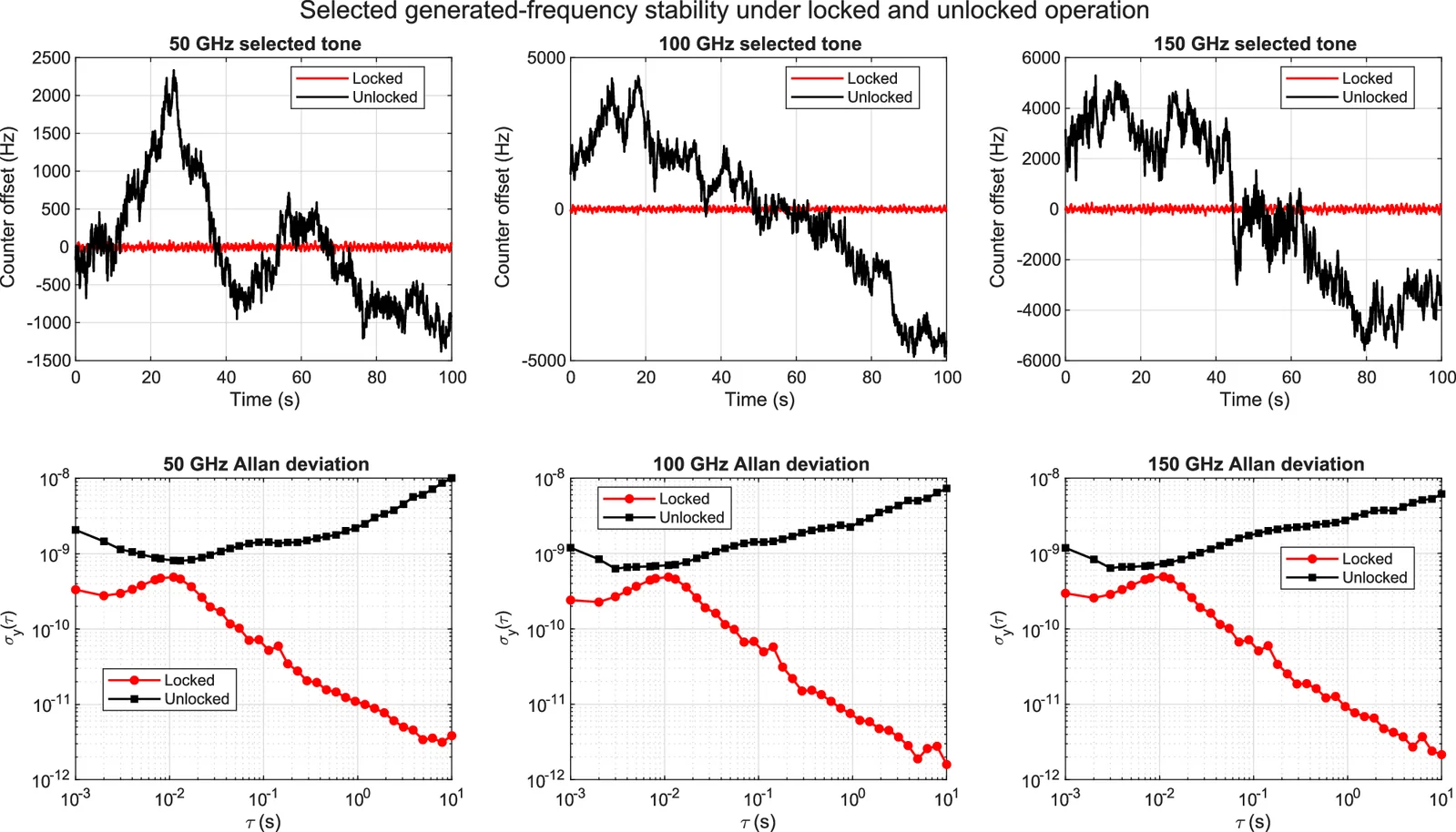
Repetition-rate-harmonic stability at 50, 100 and 150 GHz. **Top row:** counter-offset traces for selected repetition-rate harmonics at 50, 100 and 150 GHz, measured using electro-optic heterodyne/downconversion under locked (red) and unlocked (black) operation. **Bottom row:** corresponding Allan deviations, expressed relative to the selected harmonic frequency. The measurements show reduced relative frequency fluctuations under locked operation for representative harmonics of the optical-comb frequency grid.

To test whether the reference-derived stabilisation is transferred beyond the fundamental beat-note line, we measured selected repetition-rate harmonics of the feeding optical comb at 50, 100 and 150 GHz using the electro-optic heterodyne/downconversion readout described in the Methods. For each measurement, the modulation frequency was chosen so that the targeted harmonic was translated into the accessible RF beat-note band (Fig. 6). When the repetition-rate lock is engaged, the counter offsets remain close to zero, and the Allan deviations are reduced with respect to the free-running traces. This supports the interpretation that each line of the generated THz comb is seeded by the corresponding reference-stabilised repetition-rate harmonic of the optical comb.

We have demonstrated direct millimetre-wave baseband comb generation in the sub-THz range through photoconductive conversion of a laser-cavity-soliton microcomb system. The long-term stability of the source enables operation with standard commercial THz-TDS hardware and, thanks to the high spectral efficiency of the microcomb, allows direct operation without additional amplification when using modest optical pulse power (~ 5 mW) and low electrical pump power (< 1.5 W).

The low-jitter free-running operation supports TDS with relaxed synchronisation constraints, as demonstrated over delays spanning thousands of pulses. Our results, therefore, establish a microcomb-compatible route to single-cycle, carrier-envelope-offset-free, broadband photoconductive mm-wave comb generation. The experimentally demonstrated capabilities point to realistic future directions, including broadband calibration, multi-tone spectroscopy, relaxed-synchronisation TDS architectures, and parallel mm-wave link concepts. Repetition-rate locking further shows compatibility with precision frequency control, while multisoliton states provide a simple source-level mechanism for reshaping the harmonic envelope of the generated comb. Looking ahead, improved antenna designs and closer integration with photonic and antenna platforms should further enhance conversion efficiency and system compactness.

## Methods

### Microcomb setup and time domain spectroscopy setup

The experimental system (Supplementary Fig. 4) relies on a laser cavity-soliton (LCS) generated using a four-port, high-index doped silica integrated resonator with a free spectral range (FSR) of ~ 50 GHz and a linewidth of less than 140 MHz, corresponding to a quality factor of about 1.3 million. This micro-ring resonator was embedded within a polarisation-maintaining ytterbium-erbium co-doped fibre cavity. Specifically, the ring closes the cavity between its input and drop ports, and the through port is used as a laser output. The cavity incorporates a delay line, polarisation optics, an optical isolator, and a tuneable bandpass filter with a 10 nm bandwidth. Further details on the LCS characteristics and dynamics can be found in references^66,67^. Under typical operating conditions, we obtained a soliton output power of about 5 mW. For single-soliton operation, the 980 nm EDFA pump typically requires about 700 mW and is driven at 1 A and 1.8 V. To improve the mm-wave emission performance, we added an additional spectral shaping and amplification stage. Specifically, the output from the resonator’s through-port was filtered through a bandpass filter centred at 1540 nm with a full width at half maximum of 12 nm, designed to suppress a strong unwanted mode arising from the erbium gain spectrum at 1540 nm. This filtering significantly improved SNR in subsequent THz-TDS measurements. While the filtered output already provided sufficient power for basic THz generation and detection, a second fibre amplifier was included to increase flexibility, signal dynamics, and overall SNR, boosting the average optical power to approximately 120 mW for a single-soliton state. It is important to note that this power level varies substantially with the specific soliton configuration (e.g., the number of solitons in the state). The filtered and amplified pulse train was subsequently directed into a conventional fibre-coupled THz-TDS setup. Here, a beam splitter separated the pulses into distinct pump and probe paths, with an optical delay stage controlling their relative timing. Both paths were fibre-coupled into commercial fibre-coupled photoconductive antennas (Menlo TERA15-TX-FC emitter and TERA15-RX-FC receiver). The emitted THz pulses propagated through free space and were collimated and focused using a pair of Teflon THz lenses. A photoconductive antenna generates THz transients by illuminating a biased semiconductor gap with a picosecond optical pulse, generating a transient photocurrent that radiates a THz pulse. In a common model, in the far-field, the THz electric field is proportional to the time derivative of the photocurrent:

3 $$E(t)\propto \frac{dJ(t)}{dt}.$$

The photocurrent *J*(*t*) is roughly proportional to the photoexcited carrier density *n*(*t*); a simple rate equation (*dn*/*dt* + *n*/*τ*_*e*_) ∝ *I*(*t*) describes the carrier dynamics. Here, *I*(*t*) is the optical intensity envelope, and *τ*_*e*_ is the carrier lifetime. When *τ*_*e*_ is shorter than the typical pump-pulse duration, the antenna operates in the fast-recombination regime. Considering a typical microcomb pulse duration of Δ*t* = 1/2 ps, this is the relevant regime for antennas specified for pumping at *λ* = 1550 nm, typically based on LT-InGaAs semiconductor substrates^46^. At this timescale we can neglect any delayed response; hence $J(t)\approx {{{\rm{const}}}}\times I(t)$, and the emitted field approximately follows the time derivative of the optical intensity envelope, as in Eq. (1). In the same fast-recombination regime, photoconductive sampling retrieves the convolution of the emitted THz field with the envelope of the sampling pulses, as indicated in Eq. (2). It is worth highlighting that our experimental configuration, that uses commercial photoconductive antennas typically specified for optical pumping repetition rates around 100 MHz, is notably sub-optimal for our ~ 50 GHz microcomb-driven pulse train. Firstly, by constraining the average optical power to remain within the antenna’s specified damage threshold, the energy per individual pulse (and thus the field amplitude per pulse) becomes significantly lower than what the antenna specifications assume. While partially compensated by the increased repetition rate, a typical side effect of the much shorter pulse period is the incomplete depletion of photo-generated carriers that establishes a higher conductivity background. This diminishes the achievable conductivity contrast, reducing the current gradient and thereby weakening the generated field emission. More critically, the electrical supply design of standard commercial antennas is commonly optimised for replenishing charges at the photoconductor electrodes within timescales compatible with repetition rates of about 100 MHz. At our operational repetition rates, this replenishment time constant severely constrains the peak photocurrent achievable per pulse, bounding the antenna efficiency. Remarkably, our results suggest that substantial improvements, possibly spanning several orders of magnitude, may be achievable through antenna and electrical supply optimisation specifically tailored to microcomb design.

### Microcomb stabilisation setup

To stabilise the system, active control of the cavity length was implemented using a piezoelectric fibre stretcher. This stretcher comprises of a 62 cm-long segment of the Erbium-doped fibre amplifier wound around a 6 cm diameter piezoelectric cylinder. A proportional-integral-derivative (PID) control loop (mFALC 110) regulated the voltage applied to the cylinder, thereby continuously adjusting its radius to tune the overall cavity length. The scheme for locking the soliton repetition rate is shown in Supplementary Fig. 4. A portion of the resonator’s through-port output was diverted using a beam splitter and directed into an electro-optic modulator (EOM). The EOM was operated in a high-drive regime at 8.168 GHz to generate sidebands on each soliton comb line. This drive frequency was precisely tuned such that the heterodyne beatnote between the outermost sidebands of adjacent ~ 50 GHz-spaced comb lines was ~ 20 MHz. The modulated light was then directed to two photodiodes, and the output from each was passed through a 20-21 MHz electrical bandpass filter. One filtered signal was monitored with a frequency counter, while the other served as the process variable for the PID controller, which used a 20.5 MHz signal from a Sigilent generator as its reference setpoint. The resulting error signal from the PID was amplified to drive the piezoelectric fibre stretcher, thereby closing the feedback loop and locking the repetition rate beatnote to the 20.5 MHz reference. For the selected harmonic stability measurements in Fig. 6, the same electro-optic heterodyne principle was used with the modulation frequency chosen so that the targeted 50, 100, or 150 GHz repetition-rate harmonic produced a beat note within the RF band accessible to the counter and filtering electronics. It is important to note that both the frequency counter and the signal generator were synchronised to an external GPS-disciplined reference to minimise long-term noise contributions from the reference source in the locked measurements. The overall duration of our measurements was ultimately limited by the long-term stability of this locking system. Over time, slow thermal drifts and environmental fluctuations caused the repetition rate to deviate beyond the dynamic range of the piezoelectric stretcher. When this compensation limit was exceeded, the feedback loop could no longer maintain its lock, preventing longer data acquisition times.

## Supplementary information

Supplementary Information

Transparent Peer Review file

## Data Availability

The data that support the findings of this study are openly available at https://doi.org/10.17028/rd.lboro.30272239.

## Code availability

The codes used during the current study are available from the corresponding author on request.
