# Supplementary material for: Millimetre-wave comb generated by an optical microcomb
Source: Nat Commun. 2026 Aug 20;17:9833. doi: 10.1038/s41467-026-76747-2 (PMC13578215; doi:10.1038/s41467-026-76747-2)
Supplement: Supplementary file 1 [file 41467_2026_76747_MOESM1_ESM.pdf]

Supplementary Information
Millimetre-Wave Comb Generated by an Optical Microcomb

L. Peters et al.

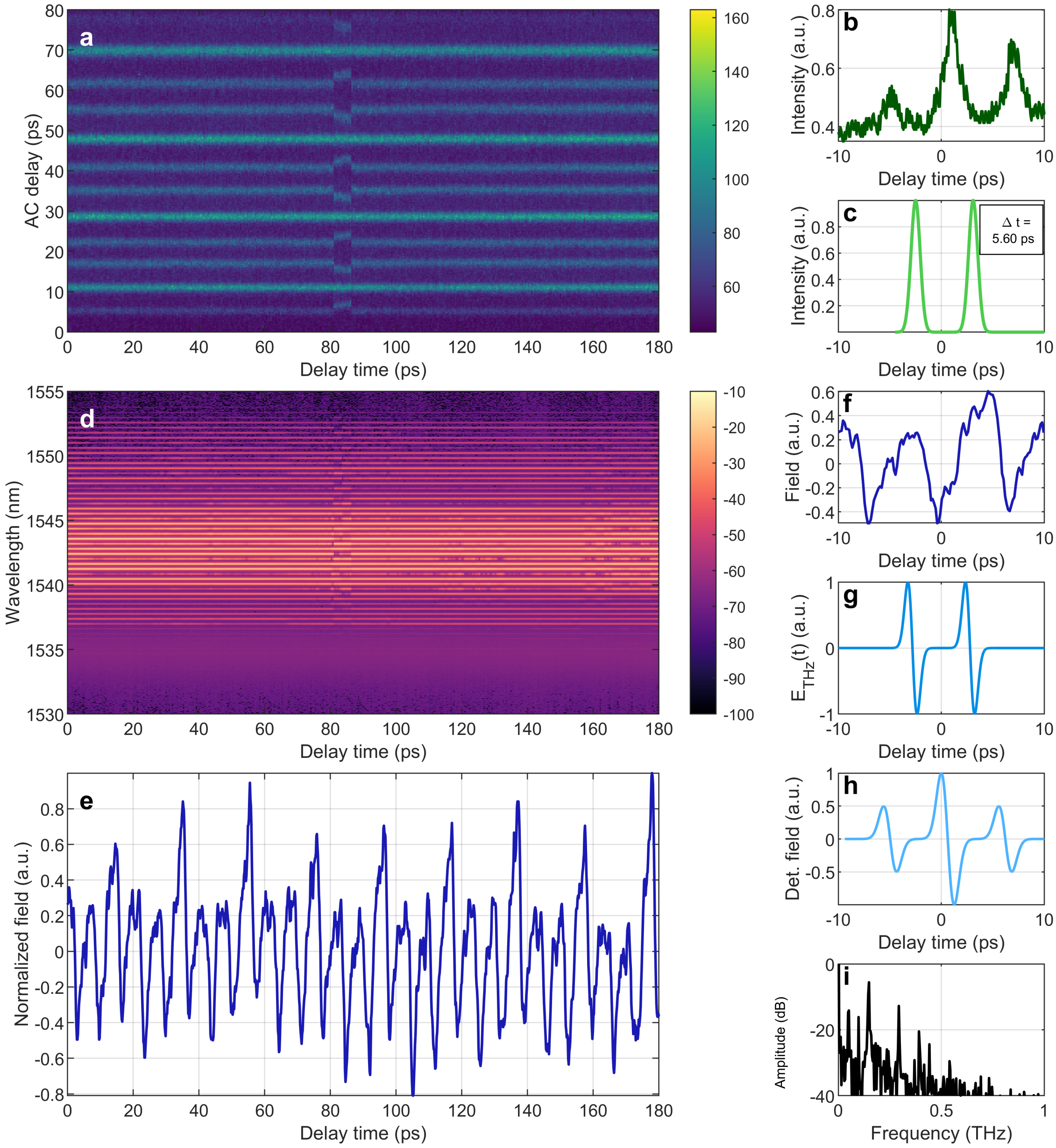

Supplementary Fig. 1: Alternate case for two-soliton long-term stability. **a** False-colour map of the autocorrelation traces in time. **b** A typical autocorrelation trace. **c** Reconstructed double-soliton state with periodicity 20 ps and a non-equidistant soliton spacing extracted from the autocorrelation trace. **d** False-colour map of the optical-spectrum-analyser traces in time. **e** Experimental terahertz time-domain spectroscopy trace. **f** Experimental TDS trace for a single period. **g** Expected generated waveform obtained by applying Eq. (1) to the optical pulses in c. **h** Reconstructed TDS trace using Eq. (2) with the generated waveform in g and the optical pulses in c. **i** Experimental normalised Fourier amplitude spectrum in dB (arbitrary units) of the detected TDS waveform.

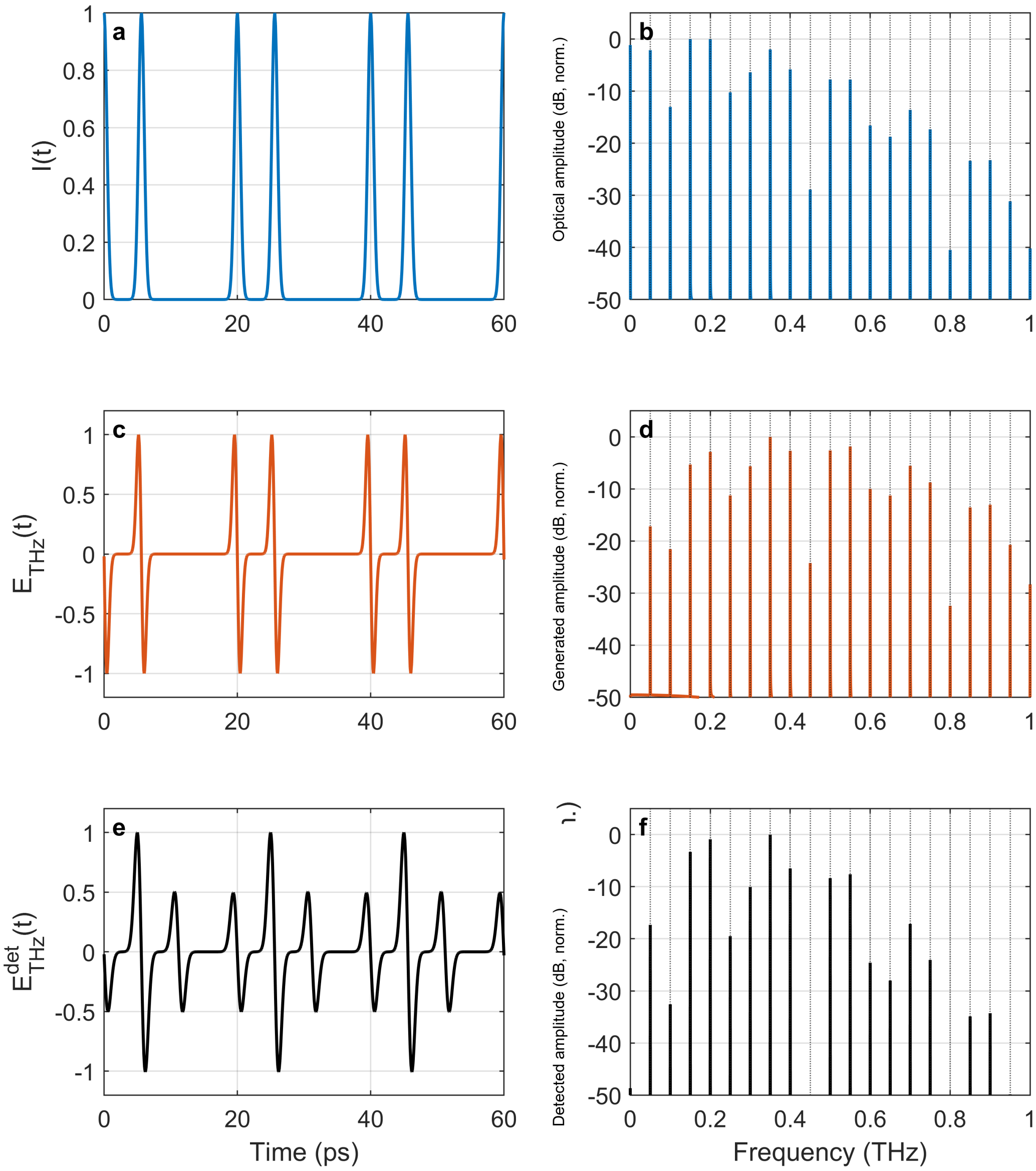

Supplementary Fig. 2: Single-case model of spectral control with a fixed soliton-pulse delay, showing the effect of optical–THz convolution in TDS reconstruction. a Normalised optical intensity over a 60 ps window. **b** Normalised Fourier amplitude spectrum in dB (arbitrary units) of the optical pulse train. **c** Normalised generated THz waveform over a 60 ps window. **d** Normalised Fourier amplitude spectrum in dB (arbitrary units) of the generated THz field. **e** Normalised detected THz waveform over a 60 ps window, obtained from the convolution of the optical intensity in a with the generated THz field in c. **f** Normalised Fourier amplitude spectrum in dB (arbitrary units) of the detected TDS field. The reduced visibility of some harmonics arises from the convolution/filtering intrinsic to the TDS detection process, not from suppression at the source.

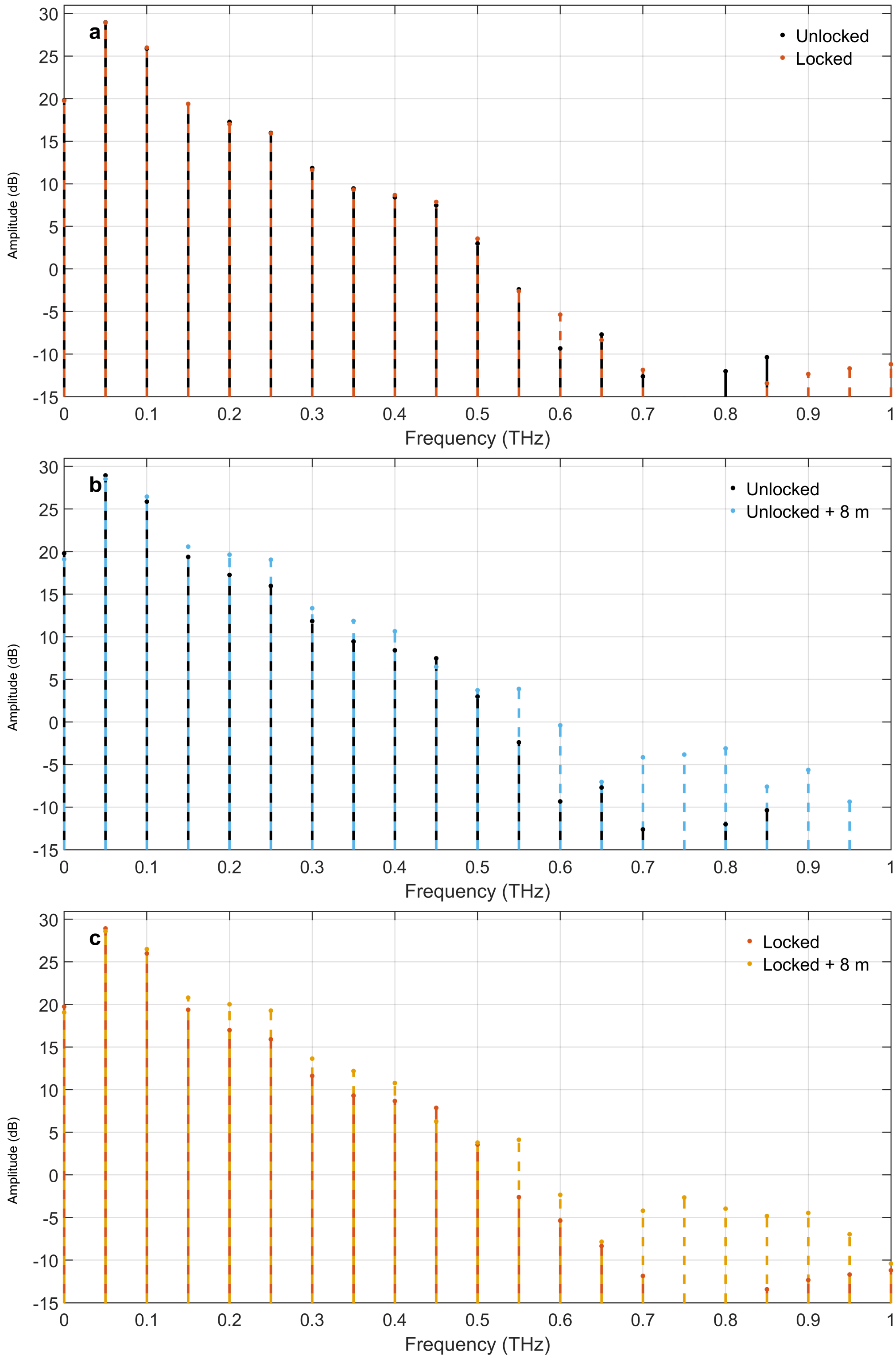

Supplementary Fig. 3: Comparison of millimetre-wave amplitude spectra under different locking conditions. The amplitude spectra corresponding to the traces shown in Fig. 5d,e are shown here. **a** Unlocked (black) versus locked (red) amplitude spectra. **b** Unlocked (black) versus unlocked with an added 8 m delay line (sky blue). **c** Locked (red) versus locked + 8 m (orange). Amplitude spectra are plotted from 0–1 THz with vertical-bar stems every 0.05 THz; dots mark the bar tips. No statistically meaningful deviation is observed after inserting the 8 m delay: residual amplitude changes are consistent with routine coupling-power drift.

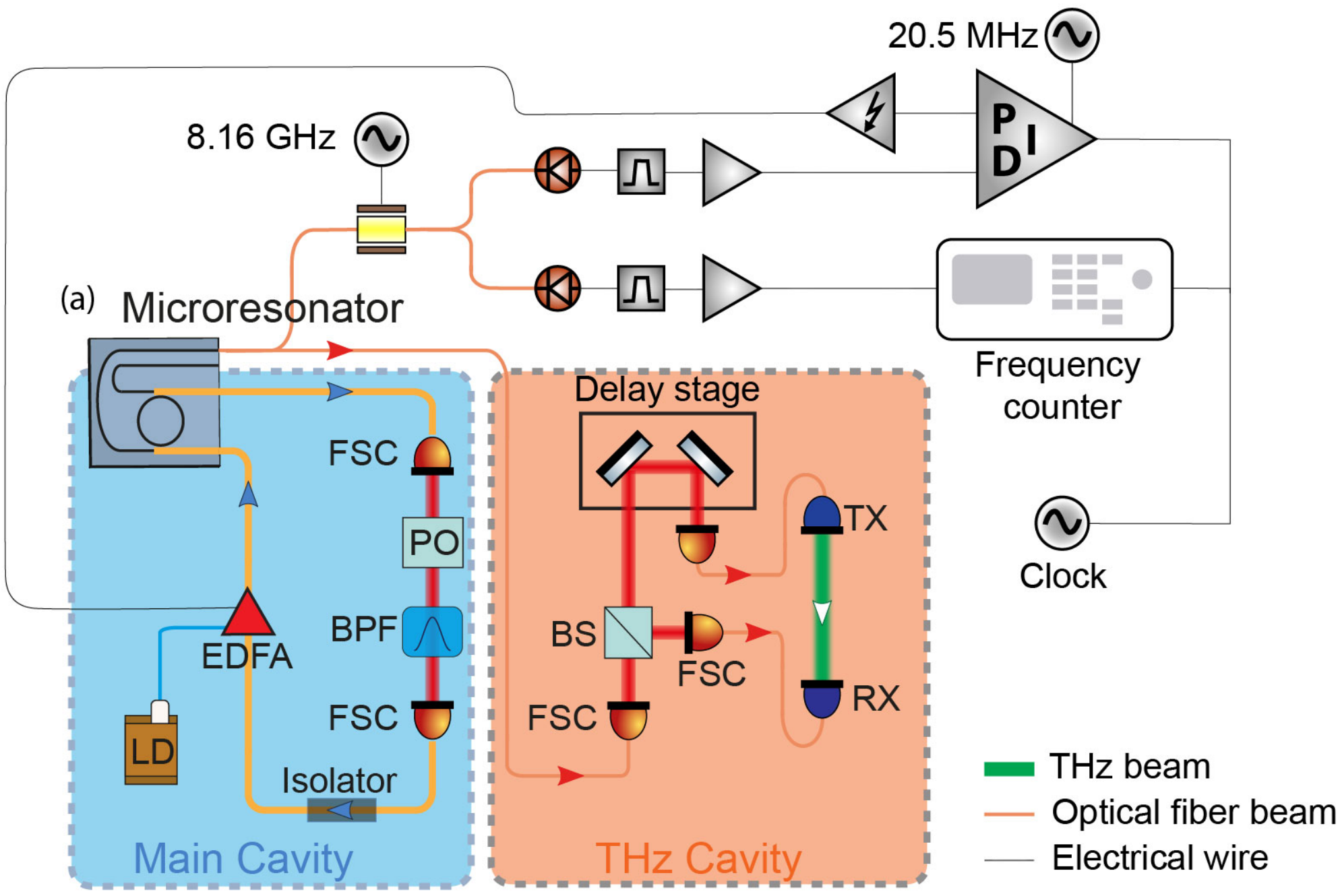

Supplementary Fig. 4: Microcomb-driven THz setup and repetition-rate lock A laser-cavity soliton is generated in a 48.9 GHz-FSR silica micro-ring that closes the main fibre cavity (blue). The cavity contains an EDFA, isolator, polarisation optics and a 10 nm band-pass filter. Light from the through port is further filtered (12 nm Gaussian at 1540 nm) and optionally re-amplified, then sent to a fibre-coupled THz-TDS stage (orange) where a beam splitter and delay line feed transmitter and receiver photoconductive antennas (TX/RX). To stabilise the repetition rate, a tap of the output is phase-modulated at 8.168 GHz; the ~ 20 MHz beat between adjacent comb lines is detected on photodiodes, filtered (20–21 MHz), and compared to a 20.5 MHz reference in a PID loop that drives a piezo fibre stretcher. All RF instruments share a GPS-disciplined clock. Colours: green—THz beam; orange—optical fibre; black—electrical. Abbreviations: LD, laser diode; EDFA, Er/Yb-doped fibre amplifier; BPF, band-pass filter; PO, polarisation optics; BS, beam splitter; FSC, fibre splitter/combiner; PD, photodiode; PID, controller.
