# Supplementary material for: Millimetre-wave comb generated by an optical microcomb
Source: Nat Commun. 2026 Aug 20;17:9833. doi: 10.1038/s41467-026-76747-2 (PMC13578215; doi:10.1038/s41467-026-76747-2)
Supplement: Supplementary file 2 [file 41467_2026_76747_MOESM2_ESM.pdf]

nature portfolio

Peer Review File

Millimetre-Wave Comb Generated by an Optical Microcomb

Corresponding Author: Dr Luke Peters

This file contains all reviewer reports in order by version, followed by all author rebuttals in order by version.

Version 0:

Reviewer comments:

Reviewer #1

(Remarks to the Author)
This manuscript presents mmwave generation by using microcomb-based time-domain spectroscopy (TDS)s, and THz output corresponding to different comb state are recorded. The stability is measured and presented in phase noise and Allan deviation. It might be the first report regarding to the THz generation by using the microcomb and photoconductive antennas, however, it is hesitating to vote this manuscript to be on Nature Communication due to the lack of intrinsic novelty and the incomplete of the presentation
Major comments:
1. After reading this manuscript, it is hard to realize what the novelty by using microcomb and THz antennas, comparing to other very well-developed approaches, either in terms of stability, size weight and power, system simplicity, or something else. Authors give a sort of plain statement in the introduction. What is the status of the technique demonstrated here in the community of comb-based THz generation? As one example, the figure 5 in the NC 14 790 (2023) could give author some hints. Overall, the novelty is somehow vague, considering the publication on Nature Communication
2. Another information missing here which might impact the novelty is the application of the technique. For example, this THz source can give out higher quality imaging than others?
Other comments
1. "metrological-grade optical soliton source". Since there is no metrology application demonstrated in this manuscript, this expression is not convinced.
2. Figure 1a, should label all devices shown here.
3. Figure 1 b, d and e. The PSD unit is incorrected. Caption d, PSD is not Power spectrum. Seem like authors measure power spectrum, and the unit should be dBm. Same incorrect in figure 2d, 3i, A1 i, A3
4. There is no content related to figure 1 d and e, except captions
5. Figure 5. According to the method 4.2 and figure 4a, the comb performance after the frequency stabilization, red lines in figure 5a, 5b and 5c, are "inloop" performance. From figure 5, the conclusion the residual error of the servo which can lock the comb repetition. To support the statement "This value reflects the intrinsic stability of the microcomb, allowing direct assessment of the system's long-term performance", the out-of-loop measured is needed.
6. Page 7, the paragraph should be separated when discussion moves from figure 4 to figure 5.

Reviewer #2

(Remarks to the Author)
The manuscript 'Millimetre-Wave Comb Generated by an Optical Microcomb' by Peters et al., reports on the direct generation of millimetre-wave baseband frequency combs via photoconductive conversion of an optical microcomb. The topic is central to current efforts at the interface of microcomb physics, millimetre-wave/THz generation, and precision measurements. The experimental approach is clearly defined, and the results are supported by an extensive and coherent characterisation.

A central result of the paper is the demonstration of a true baseband millimetre-wave frequency comb obtained directly from a soliton microcomb, which is enabled by the specific efficiency properties of the microcomb. This is an important innovation with respect to existing work on microcomb-based mm-wave and THz generation, which has so far predominantly focused on single-frequency carriers or narrowband signals. In contrast, the present approach enables direct access to a broadband mm-wave comb that is intrinsically coherent, carrier-envelope-offset free and extends into the sub-THz regime, which has a key impact on mm-wave signal distribution.

The use of multisoliton states as a mechanism for shaping the millimetre-wave spectral output represents another distinctive aspect of the work. The manuscript demonstrates that the relative temporal separation of solitons in the optical domain directly translates into controlled redistribution of spectral power in the generated mm-wave comb (Figs. 3 and 4). This strategy relies on intrinsic microcomb dynamics rather than external filtering or electronic post-processing, and provides a physically transparent route to spectral tailoring within the same platform.

The manuscript is generally well structured and clearly written. I have some minor remarks which the authors can address to strengthen their presentation:

1- While application areas are mentioned throughout the introduction, the link between specific experimentally demonstrated capabilities and concrete use cases could be articulated more explicitly. For example, the work demonstrates baseband operation (Fig. 1d-e), carrier-envelope-offset-free comb generation, long-delay coherence (Fig. 5e), and spectral shaping enabled by multisoliton states (Figs. 3-4). Briefly mapping these features onto representative application scenarios, such as broadband calibration sources, multi-channel spectroscopy, or parallel mm-wave links, in a dedicated paragraph at the end of the introduction or in the conclusions would improve contextual clarity.

2- The positioning with respect to previous microcomb-based mm-wave approaches could be sharpened in the introduction section. In particular, when discussing earlier work based on optical division and photodiode technologies (refs. [14-17], [58-65]), it would be useful to state more explicitly that those approaches primarily address carrier or single-frequency signal generation, whereas the present work targets direct generation of a broadband baseband comb. Making this distinction explicit would help clarify the conceptual advance introduced here.

3- In Section 2, the description of the photoconductive TDS process is accurate, but the consequences of the convolution inherent to Eq. (2) could be stated more precisely in the main text. In particular, I would advise that the statement saying that the harmonics are somewhat suppressed in the discussion of Figs. 3f-h is rather vague, and it would benefit from a more explicit explanation. Clarifying the physical origin and extent of this suppression, by explicitly attributing it to the temporal convolution imposed by the TDS detection scheme, rather than to the microcomb source itself, would improve technical clarity and reduce potential ambiguity.

4- In the discussion of repetition-rate stabilisation (Fig. 5), the manuscript notes that locking does not significantly affect the observed mm-wave comb properties. It would be helpful to more explicitly relate this observation to the finite temporal window of the TDS scan (approximately 500 ps), which limits the effective spectral resolution to the GHz scale and renders the measurement largely insensitive to long-term stabilisation effects. Introducing this point slightly earlier would improve the internal logic of the discussion.

Editorial points and typos:

- Section 4.1: The EDFA 980 nm pump for single soliton operation is typically requires 700 mW contains a grammatical duplication.

- Figure 1 caption: (/sim 50) GHz should likely read (~50) GHz.

- Page 9: show ? shown.

- Section 2 (discussion around Fig. 3): showin in in Fig. 3f.

- Consistency between millimetre-wave and millimetre-wave spelling should be checked to conform with the journal style.

To conclude, the manuscript presents the first experimental proof of a millimetre-wave comb from a chip-based microcomb, which is significantly above the state of the art and fits a wide international effort on the topic. After addressing my minor comments, I strongly suggest its publication in Nature Communications.

Version 1:

Reviewer comments:

Reviewer #1

(Remarks to the Author)
Thanks for the revised versions with further discussion or explanation. I do not have further questions, and recommend this manuscript to be published.

Reviewer #2

(Remarks to the Author)
I thank the authors for their detailed responses and for the revisions made to the manuscript. I also note the substantial effort devoted to addressing the concerns raised throughout the review process. The revisions satisfactorily address the points

raised in my previous review.

In particular, the manuscript now provides a clearer link between the demonstrated experimental capabilities and representative application scenarios, which improves the overall context of the work.

The discussion of the relationship to prior microcomb-based mm-wave approaches has also been clarified, with a more explicit distinction between previous single-carrier and optical-division techniques and the broadband baseband comb approach presented here.

The additional discussion of the photoconductive TDS process helps clarify the interpretation of the measured spectra, particularly through the improved explanation of convolution-induced filtering effects. The revisions to the discussion of repetition-rate stabilisation are also appropriate and further enhance the clarity and technical rigor of the manuscript.

Overall, I am satisfied that the comments raised in my previous review have been adequately addressed, and I have no further concerns. I therefore support publication of the revised manuscript.

Open Access This Peer Review File is licensed under a Creative Commons Attribution 4.0 International License, which permits use, sharing, adaptation, distribution and reproduction in any medium or format, as long as you give appropriate credit to the original author(s) and the source, provide a link to the Creative Commons license, and indicate if changes were made.
In cases where reviewers are anonymous, credit should be given to 'Anonymous Referee' and the source.
The images or other third party material in this Peer Review File are included in the article’s Creative Commons license, unless indicated otherwise in a credit line to the material. If material is not included in the article’s Creative Commons license and your intended use is not permitted by statutory regulation or exceeds the permitted use, you will need to obtain permission directly from the copyright holder.
To view a copy of this license, visit https://creativecommons.org/licenses/by/4.0/

We thank the Editor and the reviewers for their careful reading of the manuscript and for their constructive comments.

The revision has been prepared to address the central concerns raised during review, in particular the need to state the novelty and the stability discussion more precisely. In the revised manuscript and in the present response, we frame the work as a demonstration of direct generation of a broadband millimetre-wave baseband comb from an optical microcomb using photoconductive time-domain generation and sampling.

We also clarify that an important enabling aspect of the present implementation is the use of a laser-cavity-soliton microcomb, whose high spectral efficiency makes direct coupling to photoconductive THz emitters practical. This is a key reason why the present platform enables the direct broadband comb demonstration reported here.

We have also softened over-strong metrology language, clarified the relevance to potential applications, and revised the discussion of Fig. 5 so that the locked repetition-rate data are no longer presented as a direct THz out-of-loop validation.

All manuscript changes are shown in red. For more extensive revisions, we summarise the nature of the modifications in the present response, while the accompanying redlined manuscript provides the precise location and wording of the corresponding textual changes. Reviewer comments are reproduced in purple for ease of reference.

Reviewer 1

Major Comment 1

Comment: After reading this manuscript, it is hard to realize what the novelty by using microcomb and THz antennas, comparing to other very well-developed approaches, either in terms of stability, size weight and power, system simplicity, or something else. Authors give a sort of plain statement in the introduction. What is the status of the technique demonstrated here in the community of comb-based THz generation? As one example, the figure 5 in the NC 14 790 (2023) could give author some hints. Overall, the novelty is somehow vague, considering the publication on Nature Communication

Response: We thank the reviewer for giving us the opportunity to clarify this point. We appreciate that the novelty was not stated sharply enough in the submitted version, and that this made the manuscript appear to propose a general comparison with established THz source platforms.

In the revised manuscript, we now state the advance more precisely. The central contribution is not that the present system is universally superior to other THz sources on every metric. Rather, it is the direct generation of a broadband baseband millimetre-wave comb from an optical microcomb using photoconductive time-domain generation and sampling, with multiple mutually coherent harmonics

directly observed in the converted output. We now distinguish this explicitly from prior microcomb-based work, which has predominantly focused on optical-frequency-division oscillators, photodiode-based narrowband microwave or mm-wave carrier generation, or selected-frequency photomixing, rather than a directly observed broadband baseband comb.

We also clarify why the present implementation is enabling in this context. An important aspect of the work is the use of a laser-cavity-soliton microcomb, whose high spectral efficiency, i.e. the distinctive lack of a strong non-pulsed background for the specific soliton state, makes direct broadband coupling to photoconductive THz emitters practical. This differs from more conventional Kerr-microcomb implementations, where lower spectral efficiency and the presence of a stronger CW background can make direct broadband photoconductive coupling less practical. In this sense, the novelty is not only the observation of the broadband converted comb itself, but also the identification of a microcomb platform that is compatible with this mode of operation.

We further clarify that photoconductive time-domain generation and sampling are instrumental to the present demonstration, because they give direct access to the broadband time-domain waveform generated from the microcomb, rather than only to a narrowband carrier or selected beat note, as in the seminal literature. This TDS-style configuration enables the observation of a converted output containing multiple mutually coherent harmonics, as well as the tracking of spectral-envelope changes reshaped through transitions between multisoliton states.

Changes made in manuscript:

> Rewrote the final part of the Introduction to state the novelty as direct generation of a broadband baseband comb using photoconductive time-domain generation and sampling, rather than a general claim of best overall source performance. Revised the Abstract and Conclusions so that the positioning is aligned with this narrower and more defensible novelty claim.

Major Comment 2
Another information missing here which might impact the novelty is the application of the technique. For example, this THz source can give out higher quality imaging than others?

Response: We agree that the application relevance needed to be stated more clearly, and we also agree that the manuscript should not imply application superiority that was not demonstrated.

In the revision we now distinguish explicitly between demonstrated capability and plausible future application. The manuscript demonstrates direct baseband comb generation with multiple harmonics, carrier-envelope-offset-free operation, long-delay coherence compatible with TDS, and source-level spectral redistribution through multisoliton states. We now map these experimentally demonstrated features onto realistic application directions such as broadband calibration, multi-tone spectroscopy, coherent source architectures and parallel mm-wave links.

However, we do not claim superior imaging quality, superior communication performance, or superior end-use performance relative to alternative THz sources, because those comparisons were not part of the present study.

Changes made in manuscript:

We have added a dedicated application-relevance paragraph at the end of the Introduction, now stating:

Here, we demonstrate direct generation of a broadband millimetre-wave baseband comb in the sub-THz regime from an optical microcomb. In contrast to previous microcomb-based mm-wave approaches, which have primarily targeted single-frequency carriers through optical division, photodiode-based generation, or selected-frequency photomixing \cite{Zhang2019Terahertz,Lucas2020Ultralow,Kwon2022Ultrastable,Sun2024Integrated,Kudelin2024Photonic,Zhao2024All,Tokizane2023Terahertz,Jia2022Integrated}, the present work accesses a directly observed broadband baseband comb with multiple mutually coherent harmonics in the converted output. Photoconductive time-domain generation and sampling are instrumental in this context because they provide direct access to single-cycle, carrier-envelope-offset-free waveforms in a TDS-style configuration, rather than only to a narrowband carrier. A key enabling aspect of the present implementation is the use of a microresonator-filtered fibre laser operating in the laser-cavity-soliton regime (Fig.~1a,b), whose high spectral efficiency, i.e. the distinctive lack of a strong non-pulsed background for the specific soliton state, makes direct coupling to photoconductive emitters practical, including operation without additional optical amplification (Fig.~1c). We further show that multisoliton states provide a simple source-level mechanism for reshaping the harmonic envelope of the generated mm-wave comb while preserving the common line spacing (Fig.~1d,e).

We have revised the Conclusions to link the experimentally demonstrated capabilities to realistic future application directions, in a future-facing and capability-based form, without implying demonstrated superiority in imaging or communications:

\rev{We have demonstrated direct millimetre-wave baseband comb generation in the sub-THz range through photoconductive conversion of a laser-cavity-soliton microcomb system. The long-term stability of the source enables operation with standard commercial THz-TDS hardware and, thanks to the high spectral efficiency of the microcomb, allows direct operation without additional amplification when using modest optical pulse power ($\sim$ 5 mW) and low electrical pump power ($<$ 1.5 W).}

\rev{The low-jitter free-running operation supports TDS with relaxed synchronisation constraints, as demonstrated over delays spanning thousands of pulses. Our results therefore establish a microcomb-compatible route to single-cycle, carrier-envelope-offset-free, broadband photoconductive mm-wave comb generation. The experimentally demonstrated capabilities point to realistic future directions including broadband calibration, multi-tone spectroscopy, relaxed-synchronisation TDS architectures, and parallel mm-wave link concepts. Repetition-rate locking further shows compatibility with precision frequency control, while multisoliton states provide a simple source-level mechanism for reshaping the harmonic envelope of the generated comb. Looking ahead, improved antenna designs and closer integration with photonic and antenna platforms should further enhance conversion efficiency and system compactness.}

Other comments 1

“metrological-grade optical soliton source”. Since there is no metrology application demonstrated in this manuscript, this expression is not convinced.

Response: We appreciate that the wording too strong and could be read as implying a demonstrated metrology application or a metrological performance claim beyond what is shown.

We have therefore softened this language throughout the manuscript. Where appropriate, we now use terms such as 'high-stability' and formulations indicating compatibility with precision frequency control. This preserves the intended link to frequency-controlled operation without implying that a metrology use case was directly demonstrated. To further support the metrological relevance of the generated comb beyond the first harmonic associated with the microcomb repetition rate, we have also analysed additional measurements of selected repetition-rate harmonics of the feeding optical comb at 50, 100 and 150 GHz. While these measurements do not constitute a full out-of-loop validation of the entire comb, they do show that referenced stability transfer is observed on multiple representative repetition-rate harmonics from the same microcomb platform, rather than only on the fundamental repetition-rate line. We refer to the details of these measurements in our response to ‘Other comment 5’.

Changes made in manuscript:

We have removed 'metrological-grade' language from the Abstract, Introduction, and Conclusions, and more generally softened wording throughout the manuscript where it could imply a stronger metrological claim than supported by the present data. We have replaced over-strong phrasing with more precise formulations such as 'high-stability' and 'compatible with precision frequency control'.

Other comments 2

Figure 1a, should label all devices shown here.

Response:

In the revision we have expanded the Fig. 1a labelling so that the main optical, electrical and THz-path components are explicitly identified. This includes the microresonator, EDFA, filters, polarisation optics, beam splitter, delay line, and the transmitter and receiver photoconductive antennas.

Changes made in manuscript:

Updated Fig. 1a labelling for all principal components and signal paths.

Other comments 3

Figure 1 b, d and e. The PSD unit is incorrected. Caption d, PSD is not Power spectrum. Seem like authors measure power spectrum, and the unit should be dBm. Same incorrect in figure 2d, 3i, A1 i, A3

Response: We addressed the reviewer point:

These traces were obtained by Fourier transforming the measured time-domain waveforms and plotting the resulting normalised spectral amplitudes in dB. In the revised manuscript we now describe these traces as normalised Fourier amplitude spectra in dB (arbitrary units), which is a more faithful description of what is measured.

Changes made in manuscript:

Revised axis labels and captions in Figs. 1, 2, 3 and the Extended Figures to remove PSD terminology. Replaced 'power spectrum (PSD)' / 'power spectral density' with 'normalised Fourier amplitude spectrum' or 'amplitude in dB (arbitrary units)' as appropriate.

Other comments 4

There is no content related to figure 1 d and e, except captions

Response:

We addressed the reviewer point regarding the discussion of Fig. 1d,e being too compressed in the submitted version.

We have now inserted explicit text in the Results section to explain what Fig. 1d and Fig. 1e show. In particular, we now state that the single-soliton case directly reveals a comb of harmonics spaced by the microcomb repetition rate, whereas the two-soliton case redistributes the harmonic envelope while preserving the common spacing. This makes Fig. 1 function as a genuine entry point to the central experimental result rather than only a captioned overview.

Changes made in manuscript:

Added a new explanatory paragraph in the Results section immediately after the first discussion of Fig. 1c-e.

Other comments 5

Figure 5. According to the method 4.2 and figure 4a, the comb performance after the frequency stabilization, red lines in figure 5a, 5b and 5c, are “inloop” performance. From figure 5, the conclusion the residual error of the servo which can lock the comb repetition. To support the statement “This value reflects the intrinsic stability of the microcomb, allowing direct assessment of the system’s long-term performance”, the out-of-loop measured is needed.

Response: We understand the reviewer’s point.

We revised the Fig. 5 discussion so that it no longer states that the Allan deviation directly reflects the intrinsic stability of the emitted THz comb or constitutes a direct THz out-of-loop validation. Instead, we now describe the original Fig. 5 data as characterising the referenced repetition-rate readout and the performance of the locking scheme.

To strengthen this section, we analysed the newly supplied locked repetition-rate dataset. Here we show a referenced locked repetition-rate measurement rather than a direct THz-domain readout. The representative 100 s trace fluctuates within approximately -31 to +27 mHz around a 20.099 MHz reference (a), and the Allan deviation decreases from 2.9 x 10^-13 at 1 s to 5.5 x 10^-15 at 45 s (b).

Because the locked readout and locking electronics are referenced to the same GPS-disciplined source, values below the GPS-reference specification (a few parts in 10^{-12} over averaging times from 1 to 100 s) are interpreted as transfer coherence of the locked comb/readout path, not as an independent absolute stability measurement.

A strict out-of-loop validation below our reference level would require independent, uncorrelated metrological references, which is outside the scope of the present work.

We also analysed newly supplied measurements of selected repetition-rate harmonics of the feeding optical comb at 50, 100 and 150 GHz. These contain locked and unlocked counter traces for three representative frequencies, obtained using the electro-optic heterodyne/downconversion readout. When expressed relative to the nominal selected harmonic frequency, the locked traces give Allan deviations in the low-10^-11 range at 1 s and low-10^-12 range by 10 s, whereas the unlocked traces remain around 10^-9 to 10^-8 over the same averaging times. These measurements are sequential rather than simultaneous, and they do not constitute full-comb or direct THz out-of-loop validation. However, they do support the interpretation that each line of the generated THz comb is seeded by the corresponding reference-stabilised repetition-rate harmonic of the optical comb. We have now incorporated these additional selected-harmonic measurements into the revised manuscript as new Fig. 6.

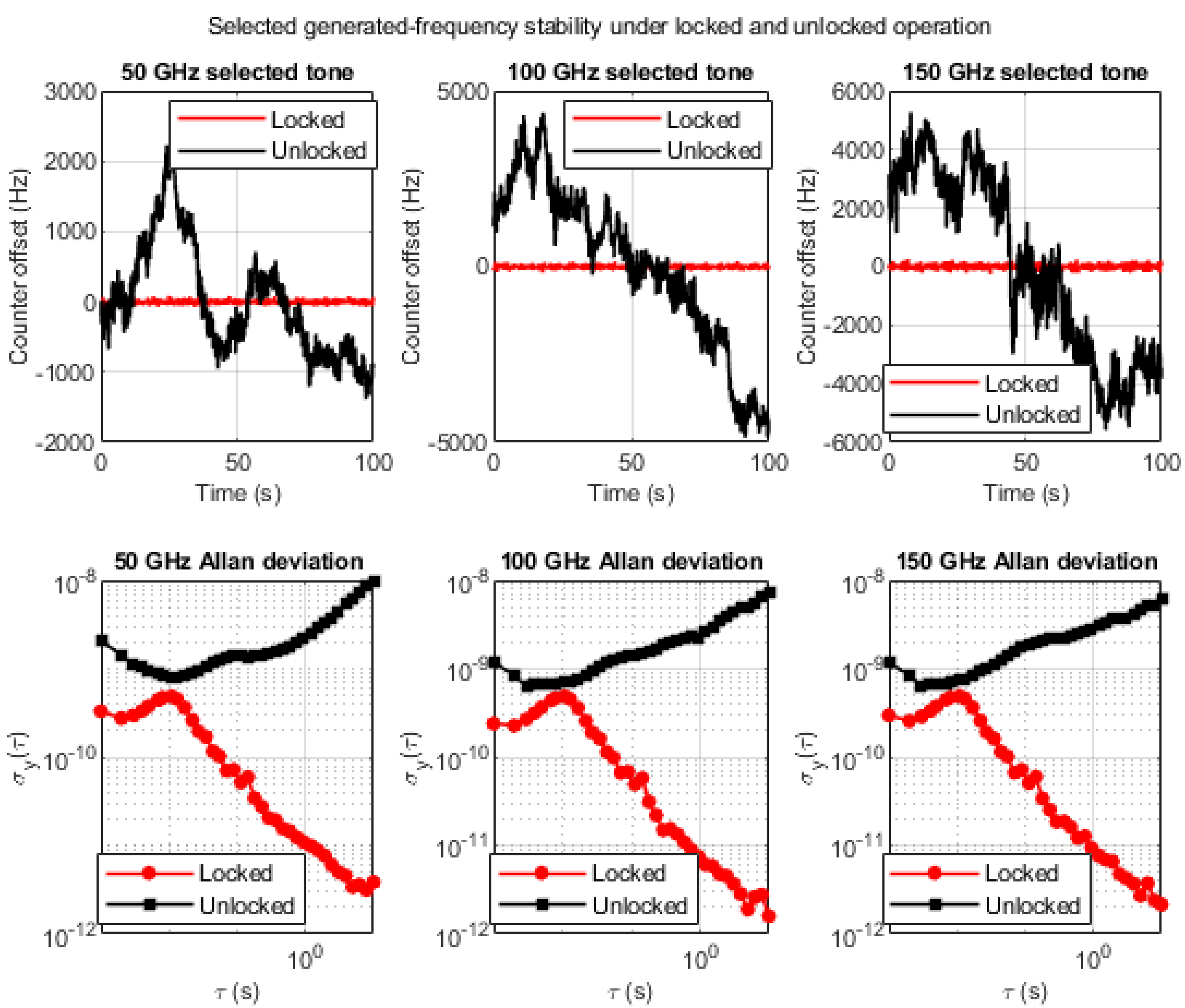

We believe this revised framing is both stronger and more accurate: the manuscript no longer hinges on an over-strong out-of-loop interpretation but instead combines a corrected lock-performance discussion with additional multi-frequency supporting data.

Changes made in manuscript:

We have removed the sentence claiming that the Allan deviation 'reflects the intrinsic stability of the microcomb'. We rewrote the Fig. 5 discussion so that it refers to referenced repetition-rate stability and lock performance, and we added the caveat that values below the GPS-reference specification should be interpreted as transfer coherence of the locked comb/readout path rather than an independent absolute stability measurement. We have updated the Fig. 5 caption to avoid out-of-loop language, and we added new Fig. 6 to the main manuscript, showing sequential electro-optic heterodyne/downconversion stability measurements of selected repetition-rate harmonics at 50, 100 and 150 GHz.

Other Comment 6

Page 7, the paragraph should be separated when discussion moves from figure 4 to figure 5.

Response: We have restructured this passage.

In the revised manuscript, the discussion of evolving soliton states (Fig. 4) is now cleanly separated from the subsequent discussion of repetition-rate stabilisation and long-delay coherence (Fig. 5), which improves both the logical flow and the readability of the section.

Changes made in manuscript:

Inserted a paragraph break and rewrote the transition between the Fig. 4 and Fig. 5 discussions.

Reviewer 2

Comment 1

While application areas are mentioned throughout the introduction, the link between specific experimentally demonstrated capabilities and concrete use cases could be articulated more explicitly. For example, the work demonstrates baseband operation (Fig. 1d-e), carrier-envelope-offset-free comb generation, long-delay coherence (Fig. 5e), and spectral shaping enabled by multisoliton states (Figs. 3-4). Briefly mapping these features onto representative application scenarios, such as broadband calibration sources, multi-channel spectroscopy, or parallel mm-wave links, in a dedicated paragraph at the end of the introduction or in the conclusions would improve contextual clarity.

Response: We thank the reviewer and appreciate that the manuscript should make this mapping more explicit.

In response, we have added a dedicated application-relevance paragraph that links direct baseband comb generation to broadband calibration and multi-tone device characterisation, long-delay coherence to relaxed-synchronisation TDS architectures, and source-level spectral redistribution through multisoliton states to multi-channel spectroscopy or parallel mm-wave links.

We also make clear in the revised text that these are realistic application directions motivated by the measurements, not claims of demonstrated end-use superiority.

Changes made in manuscript:

We have added a dedicated application-relevance paragraph at the end of the Introduction. We have revised the Conclusions so that the application discussion is capability-based and future-facing.

Comment 2

The positioning with respect to previous microcomb-based mm-wave approaches could be sharpened in the introduction section. In particular, when discussing earlier work based on optical division and photodiode technologies (refs. [14-17], [58-65]), it would be useful to state more explicitly that those approaches primarily address carrier or single-frequency signal generation, whereas the present work targets direct generation of a broadband baseband comb. Making this distinction explicit would help clarify the conceptual advance introduced here.

Response: We thank the reviewer and agree fully. This point is closely aligned with Reviewer 1's central point.

We have revised the Introduction to state more explicitly that earlier microcomb-based mm-wave and THz demonstrations largely focused on single carriers, optical-frequency-division oscillators, or selected-frequency photomixing, whereas the present work is centred on direct generation of a broadband baseband comb observable as multiple harmonics in the converted output. This distinction is now made explicitly in the prior-art positioning paragraph and in the closing paragraph of the Introduction.

Changes made in manuscript:

We have rewritten the prior-art positioning passage in the Introduction and added explicit text distinguishing the present result from single-carrier and optical-division-focused work.

Comment 3

In Section 2, the description of the photoconductive TDS process is accurate, but the consequences of the convolution inherent to Eq. (2) could be stated more precisely in the main text. In particular, I would advise that the statement saying that the harmonics are somewhat suppressed in the discussion of Figs. 3f-h is rather vague, and it would benefit from a more explicit explanation. Clarifying the physical origin and extent of this suppression, by explicitly attributing it to the temporal convolution imposed by the TDS detection scheme, rather than to the microcomb source itself, would improve technical clarity and reduce potential ambiguity.

Response:

In the revised manuscript we now state explicitly that the reduced visibility of some harmonics in the detected TDS spectra is not suppression at the source. Rather, it arises from the temporal convolution intrinsic to the TDS detection process: the emitted field is sampled by the optical pulse train, so the detected spectrum is filtered by the finite temporal structure of the sampling pulses. We also point more directly to the modelling in the Extended Figure that illustrates this effect.

Changes made in manuscript:

We have replaced the vague sentence in the Fig. 3 discussion with an explicit explanation of the convolution-induced filtering. We have revised the relevant caption wording in the Extended Figure so that the physical origin of the effect is stated more clearly.

Comment 4

In the discussion of repetition-rate stabilisation (Fig. 5), the manuscript notes that locking does not significantly affect the observed mm-wave comb properties. It would be helpful to more explicitly relate this observation to the finite temporal window of the TDS scan (approximately 500 ps), which limits the effective spectral resolution to the GHz scale and renders the measurement largely insensitive to long-term stabilisation effects. Introducing this point slightly earlier would improve the internal logic of the discussion.

Response: We have moved this explanation earlier in the discussion.

The revised text now states explicitly that pulse replicas remain mutually coherent over the approximately 500 ps TDS scan window in the free-running case, and that this coherence is further supported by clean waveform reconstruction for optical path differences as large as 8 m, corresponding to about 27 ns and over 1000 pulse periods. As a result, the longer-term low-frequency fluctuations reduced by the repetition-rate lock do not produce an observable change in the reconstructed TDS waveforms or spectral envelope over that window. Residual amplitude variations are attributed to slow power drift rather than timing decoherence between successive pulses, even though the referenced repetition-rate measurements clearly show improved long-term stability.

Changes made in manuscript:

We revised the opening of the Fig. 5 discussion to explain that the absence of observable changes in the locked and free-running TDS traces is consistent with mutual coherence of the pulse replicas over the approximately 500 ps scan window. We further clarified that the TDS reconstruction does not require alignment of the generation and detection paths to a unique zero-delay condition, because the 50 GHz repetition rate provides many equivalent pulse replicas. To support this point experimentally, we now state explicitly that clean waveform reconstruction is maintained for optical path differences as large as 8 m, corresponding to about 27 ns and over 1000 pulse periods, and that any residual spectral-amplitude variations are attributed to slow power drift rather than timing decoherence.

Comment 5

Editorial points and typos

Response: We thank the reviewer for these careful corrections. We have implemented all of them and also checked the manuscript for consistency in the spelling of 'millimetre-wave'.

Changes made in manuscript:

Corrected the duplicated grammar in the Methods section. Changed '48.91 (~50) GHz' to '48.91 GHz repetition rate (approximately 50 GHz)'. Corrected visible TeX spacing artefacts in figure references and fixed the 'millimetre-waveform' typo.
Checked spelling and hyphenation consistency throughout.

We hope that these revisions address the reviewers' concerns. The manuscript has been revised accordingly, with the novelty positioned more precisely, the application relevance clarified without overclaiming, the stability discussion made more rigorous, and the supporting analyses incorporated conservatively.
